# Predicting Quality of Life and Behavior and Emotion from Functional Auditory and Pragmatic Language Abilities in 9-Year-Old Deaf and Hard-of-Hearing Children

**DOI:** 10.3390/jcm10225357

**Published:** 2021-11-17

**Authors:** Teresa Y. C. Ching, Linda Cupples, Greg Leigh, Sanna Hou, Angela Wong

**Affiliations:** 1 Department of Linguistics, Macquarie University, Sydney 2109, Australia; linda.cupples@mq.edu.au; 2 Macquarie School of Education, Macquarie University, Sydney 2109, Australia; greg.leigh@nextsense.org.au; 3 NextSense Institute, NextSense, Sydney 2151, Australia; 4 National Acoustic Laboratories, Hearing Australia, Sydney 2109, Australia; sanna.hou@nal.gov.au (S.H.); angela.wong@nal.gov.au (A.W.)

**Keywords:** pragmatic language skills, auditory functional performance, speech intelligibility, quality of life, behavior and emotion, deaf and hard of hearing children, cochlear implants, hearing aids

## Abstract

Children who are deaf or hard of hearing (DHH) are likely to exhibit difficulties in development of psychosocial skills, pragmatic language skills, and use of hearing for social communication in real-world environments. Some evidence suggests that pragmatic language use affects peer-relationships and school engagement in these children. However, no studies have investigated the influence of functional auditory performance and use of language and speech in real-world environments on children’s behavior and emotion, and on their health-related quality of life. This study explored the relationship in DHH children at 9 years of age. Data from 144 participants of the Longitudinal Outcomes of Children with Hearing Impairment study were analyzed. Parent reports were obtained on quality of life, behavior and emotion, pragmatic language skills, and auditory functional performance of children in real life. Children’s spoken language abilities and speech intelligibility were assessed by research speech pathologists. On average, performance of children in all domains was within the range of typically developing peers. There were significant associations among functional auditory performance, use of speech and language skills, psychosocial skills, and quality of life. Multiple linear regression analyses revealed that better auditory functional performance and pragmatic language skills, rather than structural language abilities, were associated with better psychosocial abilities and quality of life. The novel findings highlight the importance of targeted intervention for improving functional hearing skills and social communication abilities in DHH children, and emphasize the importance of collaborative approaches among medical, audiology, allied health, and educational professionals to identify those at risk so that timely referral and intervention can be implemented for improving psychosocial health and well-being in DHH children.

## 1. Introduction

Past research suggests that children who are deaf or hard of hearing (DHH) are at greater risk than their hearing peers for poor psychosocial functioning [1,2,3,4,5,6] and poor quality of life (QOL: e.g., [4,7,8,9]). Exceptions have, however, been reported. Hofman et al. [10] found a small but significant advantage in self-reported QOL scores for a group of 43 adolescents with cochlear implants (CIs) as compared to their hearing peers. Martin et al. [11] found no differences in self-esteem or ability to socialize one-on-one in a small study comparing 10 five- to six-year-old CI users with 6 hearing children, and Sarant et al. [12] found little evidence at 5 or 8 years of age for a difference in psychosocial functioning in a large sample of CI users, except for a small reduction in prosocial behavior. 

Despite finding that children with HL may achieve group scores within the typical range for psychosocial skills or QOL, there is considerable within-group variability that warrants explanation, especially because effective interventions to improve psychosocial skills and QOL in this population will depend on achieving a better understanding of the variables that underlie any observed difficulties [1,13]. The aim of the current research was to shed light on this important issue.

According to the World Health Organization (WHO), quality of life (QOL) refers to an “individual’s perception of health status, psycho-social status and other aspects of life” [14] (p. 3). As one component of QOL, psychosocial status can be further divided into two domains: psychological (incorporating positive feelings; negative feelings; thinking, learning, memory and concentration; self-esteem, and body image and appearance), and social relationships [14]. Thus, in spite of their conceptual overlap and empirical correlation [4], psychosocial functioning and QOL can be distinguished and should be considered separately to highlight any differential trends in outcomes. 

Previous efforts to identify variables that might play a role in predicting psychosocial abilities and QOL have frequently used measures of structural language abilities, typically using standardized tests of morpho-syntactic skills and vocabulary knowledge. In general, findings from such studies showed that better language abilities were associated with better psychosocial abilities (e.g., [1,3,12,15,16]) and QOL (e.g., [7,8]). 

Acknowledging the empirical evidence for a positive association between psychosocial abilities and structural aspects of language use, the question motivating this research is whether functional aspects of language use might be more closely associated with psychosocial abilities and QOL. Regarding children with hearing loss who use spoken language to communicate, the functional use of language encompasses at least three important aspects: pragmatic skills, auditory performance, and speech intelligibility. Pragmatic skills can be defined as “the ability to use language in social contexts and in relationship with others as well as to understand shared meaning” [17] (p. 231); auditory performance refers to a child’s listening behavior in everyday, real-world environments; speech intelligibility refers to “the degree to which speech sounds … can be correctly identified and understood by listeners” [18]. Several past studies have examined the relationships between these three aspects, either individually or in combination, and both psychosocial abilities and QOL. 

### 1.1. Psychosocial Abilities and Functional Language Use

Most studies examining the association between functional language use and psychosocial functioning have considered the role of auditory performance and/or speech intelligibility. Leigh et al. [13] reported on the association between auditory performance and psychosocial outcomes in a large sample of 301 three-year-old children using hearing aids (HAs) or CIs who were taking part in the Longitudinal Outcomes of Children with Hearing Impairment (LOCHI) study. Two aspects of the results are noteworthy here: first, despite the children’s psychosocial scores on the Child Development Inventory (CDI; [19]) being highly variable, the group means were within the typical range for hearing children of the same age; second, better scores for language comprehension, expressive language, and auditory performance were all individually associated with better psychosocial scores. 

In two follow-up studies of children drawn from the LOCHI population two years later at five years of age, results for 356 children using HAs or CIs were reported [15,16]. The first of these studies [15], a multiple regression analysis of data from all children, showed that both structural language ability, assessed using the Preschool Language Scale—Fourth edition (PLS-4; [20]), and auditory performance, assessed using the Parents’ Evaluation of Aural/Oral Performance of Children (PEACH; [21]), accounted for significant unique variance in psychosocial functioning. In the second study [16], separate multiple regression analyses were conducted for children with CIs versus HAs. The results showed that for children with HAs, structural language ability and auditory performance were both significant predictors of concurrent psychosocial functioning, whereas for children with CIs, only auditory performance accounted for significant unique variance in psychosocial functioning once all other variables were controlled. 

Another important aspect of functional language use is speech intelligibility, which has also been associated with psychosocial functioning in children with hearing loss. For example, Freeman et al. [22] showed that although a group of school-age and teenage CI users (*n* = 51) scored within the typical range on a measure of psychosocial skills (BASC2; [23]) targeting behaviors, emotions, and adaptability, they were significantly below their typically hearing peers (*n* = 47) on all but two of the subscales (anxiety and somatization). Furthermore, better speech intelligibility was associated with fewer emotional and behavioral problems and higher scores on adaptive scales.

A more recent study to investigate the role of speech intelligibility was reported by Zaidman-Zait and Most [6], who examined the contribution of pragmatic abilities to social relationships for 33 adolescents with moderate-to-profound hearing loss and 34 with typical hearing. The participants with hearing loss used HAs or CIs and communicated with spoken language. They showed that adolescents with hearing loss had significantly greater difficulty than did their hearing peers with speech intelligibility, pragmatics, and teacher-reported peer relationship problems. Furthermore, a series of multiple regressions using data from the total participant sample revealed first, that speech intelligibility predicted self-reported school engagement, and second, that pragmatic abilities predicted teacher-reported peer relationship problems and prosocial behaviors, as well as self-reported peer supportive relationships [6]. 

In sum, past research provides evidence to suggest that better functional language use contributes to improved psychosocial functioning in children and adolescents with hearing loss. Yet to be considered, however, is the question of the relationship between functional language use and QOL.

### 1.2. Quality of Life and Functional Language Use

Functional language use has been studied less often regarding its association with the broader concept of QOL than with specific psychosocial abilities. Kushalnagar et al. [24] investigated the association between QOL and self-rated functional communication in a large, heterogeneous sample of 230 youths, ages 11 to 18 years, with hearing losses ranging from mild to profound. The results showed that when participants were grouped according to self-rated functional communication (i.e., whether they understood all, most, or some to none of their parents’ expressive communication), they differed significantly on QOL domains (including sense of self, social relationships, environment, general quality of life, participation, and self-acceptance and advocacy), with all differences favoring participants with better functional communication. Of more relevance to the current study, which focuses on children who communicate using speech, Haukedal et al. [7] reported that better functional hearing (auditory performance) was associated with better QOL in a sample of 106 CI users, ages 5.0 to 12.9 years, with the strongest association evident between auditory performance and a summary score encompassing emotional, social, and school functioning domains.

Although overall QOL was not assessed, an earlier study by Most [25] provided evidence that better speech intelligibility was associated with more positive feelings of confidence in the world and lower levels of loneliness in a small group of nine 12- to 14-year-old children with severe-to-profound hearing loss who attended regular classes within regular schools. In addition, Zaidman-Zait and Dotan [26] reported that better pragmatic abilities were associated with lower levels of perceived stress in a sample of 30 high school students with hearing losses ranging from moderate to profound who used HAs or CIs. 

In sum, studies exploring the association between functional language use and QOL are less common than those exploring the more specific link with psychosocial functioning. Although there is evidence to suggest that better functional communication [24], auditory performance [7], speech intelligibility [25], and pragmatic abilities [26] might all contribute to improvements in various measures of QOL, further research is required. What is missing from the current published literature is a study in which all three aspects of functional language use (auditory performance, speech intelligibility, and pragmatics) and structural language knowledge are examined concurrently in the same sample of participants for their associations with measures of psychosocial functioning and QOL. The aim of the current research was to fill this gap in the literature. 

### 1.3. Aims of the Current Study

The study described here examined the contribution of functional language use to measures of psychosocial status and QOL while controlling for the possible influence of structural language abilities. The participant sample was drawn from the cohort taking part in the Australian population-based study examining “Longitudinal Outcomes of Children with Hearing Impairment”, the LOCHI study [27]. Drawing on data collected for children at 9 years of age, we addressed two research questions:

1. Do nine-year-old children with hearing loss who use spoken language to communicate differ from their hearing peers in behavior and emotion and QOL?
2. To what extent does performance on tests of functional abilities and structural language abilities predict concurrent behavior and emotion and QOL in nine-year-old children with hearing loss who use spoken language to communicate?

## 2. Materials and Methods

### 2.1. Procedure

This study was carried out in accordance with the recommendations of the Australian National Health and Medical Research Council guidelines for ethical research conduct. The study protocol was approved by the Australian Hearing Human Research Ethics Committee (no. AHHREC2012-17a). Parents of child participants provided written informed consent to the protocol. 

As part of the LOCHI study battery, parents completed questionnaires that solicit information on their children’s use of language and hearing in real-world environments, behavior and emotion, and QOL at 9 years of age. Research speech pathologists completed direct assessments of spoken language skills and nonverbal IQ after children turned 9 years of age. The researchers also rated the intelligibility of speech produced by the children. All assessments of children were completed between age 9;0 (9 years; 0 months) and 9;11 (9 years 11 months). Parents provided demographic information by completing a study questionnaire. Audiological information was obtained through chart review, with permission from parents. All information regarding hearing threshold levels and device was current within 6 months of direct evaluation of children, and at a time closest to the actual evaluation for each child. 

### 2.2. Participants 

Participants in the LOCHI study were included in the current analyses if they completed direct assessments of spoken language and nonverbal IQ at 9 years of age, and their parents completed questionnaires on pragmatic communication skills, auditory functional performance, behavior and emotion, and quality of life of their children at 9 years of age. Data on measures of 144 children were included in this report. All participants receive hearing services from the government-funded national hearing service provider in Australia, at no cost to families. All children use HAs or CIs. Table 1 provides descriptive statistics of the demographic characteristics of the current sample.

### 2.3. Predictor Measures: Language Assessments, Nonverbal Cognitive Ability, Pragmatic Skills, Auditory Functional Performance, and Speech Intelligibility

#### 2.3.1. Spoken Language Ability

Children’s spoken language ability was directly assessed by research speech pathologists using the Clinical Evaluation of Language Fundamentals—4th Edition (CELF-4; [28]). The CELF is a standardized test of spoken English. It includes verbal tasks that enable children to demonstrate understanding of and ability to produce English language structures. It gives an overall core language score and two subtest scores—receptive language and expressive language. 

#### 2.3.2. Nonverbal Cognitive Ability 

The Wechsler Nonverbal Scale of Ability (WNV; [29]) was administered directly to children. The WNV is a standardized test of nonverbal cognitive ability. It gives a full-scale IQ score. 

#### 2.3.3. Pragmatic Use of Spoken Language 

Pragmatic language use was assessed by parental report using the Children’s Communication Checklist—Second Edition (CCC-2; [30]). This checklist has been validated for screening communication problems in children aged 4 to 16 years [31]. The checklist consists of 70 statements of behavior. These items describe behaviors across 10 domains: speech, syntax, semantics, coherence, inappropriate initiation, stereotyped language, use of context, nonverbal communication, social relations, and interests. In response to each item, respondents rate how often they observe the behaviors in their child in real life. Ratings are made on a four-point scale: less than once a week (or never), at least once a week, once or twice a day, or several times a day (or always). Scores are expressed as scaled scores for each domain. The scores on the first 8 domains were used to derive a General Communication Composite (GCC) score. 

#### 2.3.4. Auditory Functional Performance

Auditory functional performance in real life was assessed by parental report using the Parents’ Evaluation of Aural/Oral Performance of Children (PEACH; [21]). The scale was designed to assess children’s listening and communicative behavior in real-world environments, with published data showing good reliability and sensitivity. Normal values for typically developing children and for children with hearing loss are available [21,32,33]. The scale has been validated for assessing audibility and functional auditory performance of children with hearing loss [32,34]. The questionnaire contains 13 questions, two of which relate to how often a child uses hearing devices and whether the child shows discomfort in response to loud sounds. These items provide background information about hearing device usage and are not included in scoring. The remaining 11 questions ask parents to rate how often they observe the described behaviors (e.g., follow a simple verbal instruction) in real-life situations that are quiet (5 items) or noisy (6 items). Each item is rated on a five-point scale: never (0%), seldom (1–25%), Sometimes (26–50%), often (51–75%), and always (>75% of the time). The scale gives two subscale scores, quiet and noise, and an overall score. 

#### 2.3.5. Speech Intelligibility

The intelligibility of speech produced by children was rated by research speech pathologists using the Speech Intelligibility Rating scale (SIR, [35,36]). The SIR is widely used for rating how easy or hard it is to understand speech produced by DHH children [36,37]. Ratings are made on a 6-point scale: always understand the child with little or no effort (1), almost always understand but need to listen carefully (2), typically understand about half of the child’s speech (3), typically understand about 25% of the child’s speech (4), very hard to understand (5), or almost never understand the child’s speech (6). 

### 2.4. Dependent Measures: Behavior and Emotion and Health-Related Quality of Life 

#### 2.4.1. Behavior and Emotion

To assess children’s behavior and emotion difficulties, parents were asked to complete the Strengths and Difficulties Questionnaire (SDQ; [38]). The SDQ is a widely used behavioral screening measure to identify behavior and emotional problems in children, with published data on factor structure, internal consistency, and reliability [39]. It has been recommended as suitable for use with DHH children [40]. This instrument comprises items in each of five subscales: conduct problems (e.g., “often fights with other children or bullies them), hyperactivity (e.g., “easily distracted, concentration wanders”), emotional symptoms (e.g., “many fears, easily scared”), peer problems (e.g., “picked on or bullied by other children”), and prosocial behavior (e.g., “considerate of other people’s feelings”). Each subscale consists of five items. Each item is rated on a 3-point response scale: 0 = “not true”; 1 = “somewhat true”, and 2 = “certainly true”. Scores from the first four domains (excluding prosocial behavior) were summed to make a “total difficulties score”. Higher scores on the four domains and total difficulties reflect difficulties, whereas higher scores on the prosocial domain reflect strengths. Australian normative data by age group (7–10 years) and gender [41] were used to calculate z-scores. All “difficulties” scores were reversed so that higher z-scores reflect less problems. 

#### 2.4.2. Health-Related Quality of Life

The measure of health-related quality of life (HRQOL) focuses on children’s well-being and functionality in real-world environments, as perceived by parents. The Pedatric Quality of Life Inventory version 4.0 Generic Core Scales were designed to measure the core dimensions of health [14] as well as role (school) functioning (PedsQL 4.0; [42]). The questionnaire has good reliability and validity [43], and it has been used widely in assessing QOL of DHH children [44,45]. The inventory comprises 23 items from four domains: physical health (e.g., “I have problems with running”; 8 items), emotional functioning (e.g., “I feel sad”; 5 items), social functioning (e.g., “Other children are teasing me”; 5 items), and school functioning (e.g., “It is difficult to pay attention in class”; 5 items). Each item is rated on a 5-point Likert scale: 0 = never a problem; 1 = almost never; 2 = sometimes a problem; 3 = often a problem; 4 = almost always a problem. Items were reversed-scored and rescaled to a 0–100 scale, where higher scores indicate better QOL. For scale and total scores, the mean was computed as the sum across all items divided by the number of items answered. A psychosocial health summary score was calculated as the mean score over the items answered across the emotional, social, and school functioning scales.

### 2.5. Statistical Analysis 

Descriptive statistics were used to describe characteristics of the study sample, and to summarize performance on all measures. Associations among scores were examined using Pearson’s r or Spearman’s correlation (rho) as appropriate. Multiple regression analyses were performed using total and subscale scores on the PedsQL and the SDQ as dependent variables in separate models. Age at intervention, hearing loss, nonverbal IQ, receptive language, expressive language, pragmatics (GCC score), functional auditory performance (PEACH score), and speech intelligibility (SIR score) as independent variables and device (HA or CI) as a categorical variable were used to determine the extent to which these variables predicted QOL or behavior and emotion outcomes. The normality of distribution of residuals was checked using the Kolmogorov–Smirnov test. All statistical analyses were carried out using Statistica version 10.0 [46]. A Type I error rate of α = 0.05 (two-tailed) was adopted for all statistical analyses.

## 3. Results

### 3.1. Outcomes of DHH Children Compared to Norms 

#### 3.1.1. Functional and Language Outcomes

Table 2 shows the descriptive statistics for scores on nonverbal IQ, spoken language skills, pragmatic language skills, auditory functional performance, and speech intelligibility ratings of DHH children. On average, the scores were within the range of population norms. 

#### 3.1.2. Behavior and Emotion 

**Figure 1:**
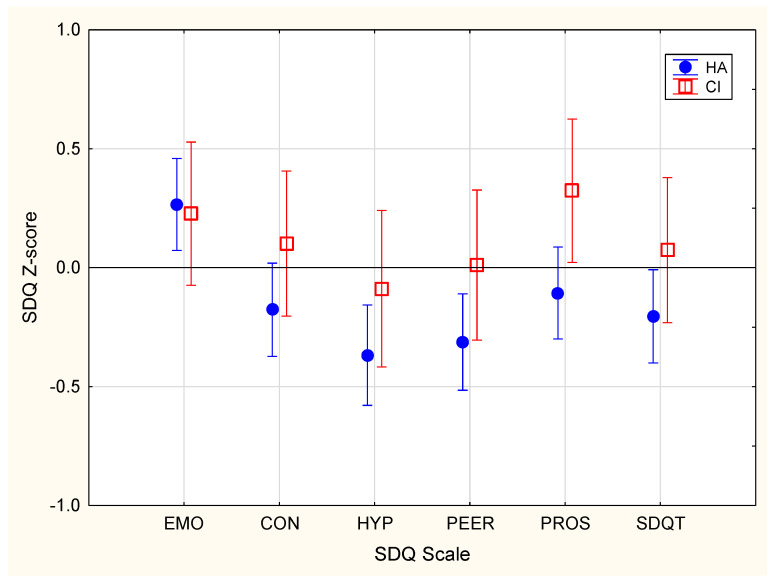
Mean Strengths and Difficulties Questionnaire (SDQ) Z-scores for children using hearing aids (HA, filled circles) and for those using cochlear implants (CI, open squares). Vertical bars indicate 95% confidence intervals. Emotion (EMO), conduct (CON), hyperactivity (HYP), peer problems (PEER), prosocial (PROS), and total difficulties (SDQT) for the SDQ are shown.

**Figure 2:**
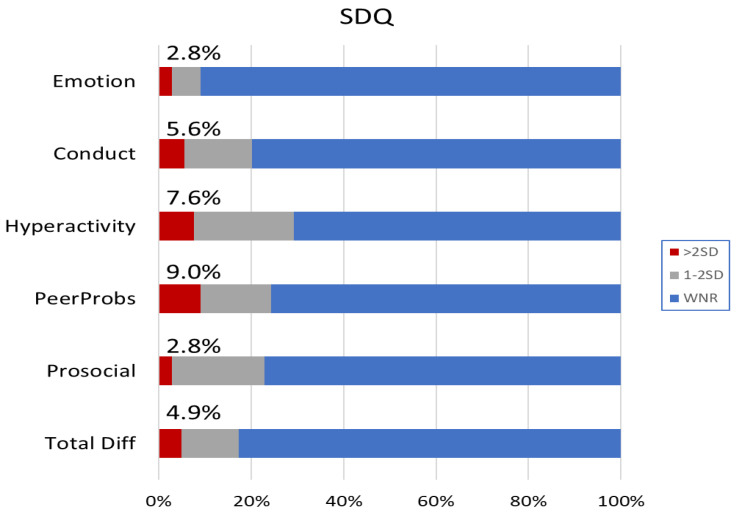
Proportion of children falling below one and two SDs of the normative mean on the Strengths and Difficulties Questionnaire (SDQ). The vertical axis shows the respective scales of the SDQ. Proportion within normal range (WNR) is shown in blue, and that below two SDs in red. The percentage values denote the proportions below two SDs for the respective scales.

Figure 1 shows the mean total and subscale scores on the SDQ, separately for children using HAs or CIs. On average, performance was within the normal range. Notably, however, a much greater than expected proportion of DHH children had scores that were below two standard deviations (SDs) of the mean for the normative population (see Figure 2). Overall, 4.9% had total difficulties scores below two SDs of the norm, compared to 2.3% expected in the normal population. 

#### 3.1.3. Quality of Life 

**Figure 3:**
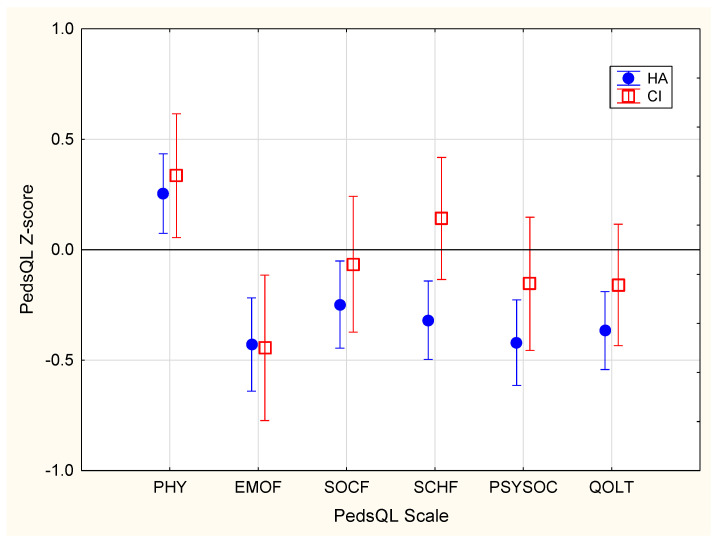
Mean Pediatric Quality of Life (PedsQL) Z-scores for children using hearing aids (HA, filled circles) and for those using cochlear implants (CI, open squares). Vertical bars indicate 95% confidence intervals. Physical health (PHY), emotional functioning (EMOF), social functioning (SOCF), school functioning (SCHF), psychosocial health (PSYSOC), and total (QOLT) on the PedsQL are shown.

**Figure 4:**
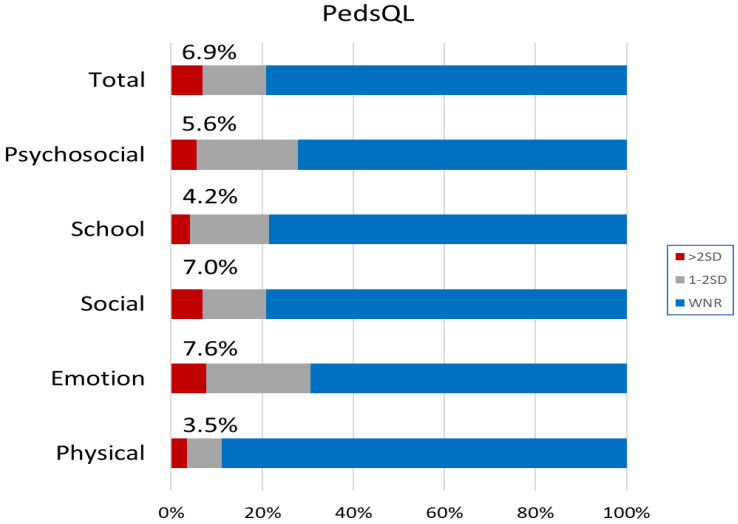
Proportion of children falling below one and two SDs of the mean on the Pediatric Quality of Life Inventory (PedsQL). The vertical axis shows the respective scales of the PedsQL. Proportions within normal range (WNR) are shown in blue, and those below two SDs in red. The percentage values denote the proportions below two SDs for the respective scales.

Figure 3 shows the mean total and subscale scores on the PedsQL, separately for children using HAs or CIs. Z-scores were derived using published norms for the instrument. On average, DHH children achieved scores within the normal range. As with the SDQ, however, Figure 4 shows that a much greater than expected proportion of children had scores that were below two SDs of the mean for the normative population. Overall, 6.9% had total scores below two SDs of the normal population. 

### 3.2. Factors Influencing Behavior and Emotion, and Quality of Life 

#### 3.2.1. Correlations among Measures

Table 3 gives the pairwise associations of performance on the PEACH, CCC-2, and each of the subscales of the SDQ and the PedsQL; together with age at intervention, hearing loss, and nonverbal IQ. Age at intervention was significantly related to spoken language ability and pragmatic language skills as well as prosocial behavior on the SDQ. The negative coefficients suggest that earlier age at intervention was associated with better structural language and pragmatic language skills as well as demonstrating more prosocial behavior. The degree of hearing loss was significantly related to school functioning on the PedsQL, and conduct, prosocial behavior, and total scores on the SDQ. The positive correlations suggest that difficulties increased as hearing loss became more severe. Higher nonverbal IQ was associated with better structural language skills, pragmatic language skills, and QOL, lesser behavior and emotion difficulties, and stronger prosocial skills.

There were significant correlations between pragmatic language skills and all subscale and total scores on the PedsQL and SDQ. Better pragmatic skills were related to less behavioral difficulty and better QOL. Better structural language skills were associated with better psychosocial skills, less behavioral difficulty, and better QOL. Children who had higher scores for functional auditory performance (PEACH) also had higher scores for structural language abilities (ReLang and ExLang), pragmatic skills (GCC), speech intelligibility (SIR), less behavioral difficulty (SDQ), and better psychosocial health and QOL. Moreover, children who produced speech that was highly intelligible also had better QOL, less behavioral difficulty, and stronger prosocial skills. Within each of the scales on the PedsQL and SDQ, there were significant correlations. Across the two measures, the subscale scores and total scores were also significantly correlated.

#### 3.2.2. Behavior and Emotion

Table 4 shows multiple regression analyses with the total difficulties, prosocial, and individual subscale scores on the SDQ as dependent variables in separate models. Functional auditory ability and pragmatic language abilities, together with degree of hearing loss and device, were significant predictors of the total score on the SDQ, accounting for 33.2% of total variance. Functional auditory ability was a significant predictor of emotion, conduct, peer problems, and prosocial scores. As the residual errors in the models for peer problems and prosocial behavior did not meet normality assumptions, the effect of the significant coefficient in these two models should be interpreted with caution. Pragmatic language ability was a significant predictor of the total difficulties and hyperactivity scores on the SDQ. Higher scores for functional auditory performance and pragmatic language skills were associated with better total difficulties scores on the SDQ (less difficulties). Degree of hearing loss was a significant predictor of the total difficulties, emotion, conduct, and hyperactivity scores on the SDQ. After allowing for the effects of demographic characteristics and functional abilities, receptive or expressive language scores were not significant predictors of the total or any of the subscale scores on the SDQ. 

#### 3.2.3. Quality of Life

Table 5 shows multiple regression analyses with the total, psychosocial health, physical functioning, and individual scale scores on the PedsQL as dependent variables in separate models. Functional auditory abilities and pragmatic language abilities were significant predictors of the total, psychosocial, emotional functioning, school functioning, and social functioning scores on the PedsQL. Degree of hearing loss was a significant predictor of the psychosocial score and emotional functioning score. After allowing for the effects of demographic characteristics and functional auditory and pragmatic language skills, neither receptive nor expressive language was a significant predictor of the total or any subscale scores on the PedsQL.

## 4. Discussion

The aims of this study were to (1) compare behavior and emotion and health-related quality of life in DHH children to normative data, and (2) determine the factors influencing these outcomes. 

### 4.1. Behavior and Emotion

We found that on average, outcomes on measures of behavior and emotion in nine-year-old DHH children were within one SD of the normative data (see Figure 1), suggesting that the current generation of DHH children do not show significantly more emotional and behavioral problems, as measured by the SDQ, than their hearing peers do. This finding is consistent with some previous studies (e.g., [12]) but not others (e.g., [1,2,3,4]). Children in the present study may have benefited from early detection and intervention for hearing loss. Although age at intervention was not a significant predictor of scores on the SDQ, age at intervention was significantly correlated with pragmatic language and expressive and receptive language scores (see Table 3). This agrees with findings of previous studies that showed a positive association between earlier intervention and better language outcomes [47,48]. Whereas some studies have reported an association between age at intervention and psychosocial development (e.g., [49]), others have shown that better language was linked to better psychosocial skills [12,15]. Our analyses revealed that the effect of age at intervention on psychosocial development was mediated by its effect on structural language and pragmatic language development. Earlier intervention was associated with better functional language and communication development, which in turn was related to better psychosocial outcomes. 

This study found evidence that functional auditory abilities (PEACH) and pragmatic language skills (GCC) were significant predictors of behavior and emotion, after allowing for the effects of structural language abilities and demographic characteristics. Our results showing a positive influence of pragmatic language skills on total difficulties and hyperactivity scores on the SDQ are broadly consistent with earlier studies [6,30,50]. However, our findings extend current knowledge by showing that functional auditory performance also contributed significantly to reducing children’s behavioral difficulties and increasing prosocial behavior. As Table 4 shows, the predicted change in total difficulties score on the SDQ was up to 0.5 SD (estimate: 0.33; 95% CI: 0.17, 0.50) per unit increase in PEACH score. The estimated effects were similar in magnitude for emotion and conduct. 

Nonverbal cognitive ability was a significant factor for only hyperactivity scores, but not for any of the other scale scores. Contrary to the findings of Sarant et al. [12] showing that more intelligent children with CIs had more peer problems and demonstrated less prosocial behavior, we found that children with higher IQ had better pragmatic skills and were reported to demonstrate less hyperactivity behavior. Our findings lend support to previous studies showing an association between deficits in language and vulnerability to poor behavioral regulation [1,51,52]. 

We observed that greater severity of hearing loss was associated with better total difficulties, emotion, conduct, and hyperactivity scores on the SDQ. This effect was likely related to the fact that almost all children with profound hearing loss in this sample used CIs. On average, children that used CIs had better total Difficulties and emotion scores on the SDQ than did those using HAs (see Figure 1), despite the latter having lesser hearing loss.

The present study found that structural language scores were not significant predictors of SDQ scores, after allowing for the effects of functional auditory performance and pragmatic language abilities. These results are consistent with findings in children with normal hearing [1], but unlike that reported in DHH children [1,12,15]. Wong et al. [15] found that language and functional auditory performance were significant predictors of psychosocial development of five-year-old children using HAs, but only auditory functional performance was a significant predictor of psychosocial development of children using CIs. Castellanos et al. [1] reported on children at 10 and 15 years of age, showing that language scores were related to adaptive skills and attention problems in children using CIs but not in normal hearing children. They suggested that language dysfluencies provide insufficient support for development of executive function, resulting in inefficient deployment of executive function, thereby affecting psychosocial outcomes. Sarant et al. [12] found that eight-year-old girls using CIs who had better receptive language had fewer conduct and peer problems. None of the previous studies, however, examined the effect of functional auditory and pragmatic language skills together with the effect of language abilities on psychosocial development. The present study showed that functional auditory skills, pragmatic language use, and structural language skills were significantly correlated (see Table 3), but the contribution of structural language skills was not significant after allowing for the effect of functional auditory and pragmatic language abilities on behavior and emotion. Although pragmatic skills are dependent on speech and structural language skills, pragmatics also encompass context-dependent comprehension, use of nonverbal skills, and broad social skills that are crucial to success in social communication and interactions in everyday environments [53]. 

Speech intelligibility was not a significant predictor of SDQ scores in the current study. This result aligns with findings of Zaidman-Zait et al. [6] that reported on teacher-rated peer relationship and prosocial scores on the SDQ. However, the current study found significant correlations between speech intelligibility ratings and the total difficulties score and the prosocial score on the SDQ (see Table 3), suggesting that children whose speech was rated as more intelligible also had fewer difficulties and demonstrated more prosocial behavior. Given that speech skills are part of pragmatic language skills, the effect of speech intelligibility was no longer significant after allowing for the effects of pragmatics and functional auditory skills. 

### 4.2. Quality of Life

On average, outcomes on health-related quality of life were within one SD of the population norm (see Figure 3) on the total and all scale scores on the PedsQL. This finding is broadly consistent with those reported in some studies [54,55,56] but not others [7,9]. Based on a meta-analysis of studies that used PedsQL, Roland et al. [9] compared DHH children with their normal-hearing peers, showing that DHH children had lower scores in school and social categories but not in physical and emotional domains. Haukedal et al. [7] reported that children using CIs (106 children aged 5–12 years) had lower scores than those of children with normal hearing on the total, psychosocial, social functioning, and school functioning scores on the PedsQL, but not on the physical health and emotional functioning scores. However, the present study shows that DHH children had QOL scores within the range of the normal-hearing population across all domains. Nevertheless, the proportion with scores at or below two SDs of the normative mean was much higher in DHH children than in the normal population (see Figure 4). This indicates that many DHH children were at risk of poor QOL. We found that about 6.9% had total scores and 5.6% had psychosocial scores below two SDs of the normative mean. Among the three scales that contributed to the psychosocial score, about 7% of children had emotion and social functioning scores and 4.2% had school functioning scores below two SDs of the population mean. 

Functional auditory performance and pragmatic language skills contributed significantly to the total and subscale scores on the PedsQL (see Table 5). Functional hearing in everyday situations (PEACH) was a significant predictor of the emotional functioning, school functioning, social functioning, psychosocial health, and total scores on the PedsQL. As shown in Table 3, PEACH scores were significantly correlated with all total and subscale scores on the PedsQL. This agrees with findings from Haukedal et al. [7] that showed significant correlations between parent report on functional hearing and PedsQL scores in children using CIs, and with findings reported by Yu et al. [57] for preschool children. The contribution of functional hearing ability to QOL was significant after allowing for the effects of other demographic characteristics and structural language abilities. 

Pragmatic language skills also contributed significantly to the total score, psychosocial summary score, school functioning, and social functioning scores on the PedsQL. As with functional hearing abilities, pragmatics skills were significantly correlated with all total and subscale scores on the PedsQL. This lends support to earlier findings by Kushalnagar et al. [24] on an association between self-rated functional communication and QOL in DHH adolescents. We found that the contribution of pragmatic skills to PedsQL scores was significant after allowing for the effects of structural language abilities. Even though receptive and expressive language scores were correlated with scores on the PedsQL, consistent with findings reported in some studies [7], these effects were no longer significant in the final model. These results suggest that although morphosyntactic and vocabulary skills are an integral part of pragmatic language skills, the cognitive, social, and nonverbal aspects of pragmatic skills significantly contributed to improving QOL beyond the effect of structural language abilities. This is a novel finding as previous studies that reported an important role for structural language abilities did not consider the concurrent effects of pragmatic language skills and functional hearing abilities. 

As was the case for the SDQ results, speech intelligibility was not a significant predictor, after allowing for the effects of pragmatic language abilities and functional auditory performance and other variables. 

Age at intervention was not significantly correlated with scores on the PedsQL, consistent with findings in recent studies that included children who received early intervention [7]. Nevertheless, it is noteworthy that the correlations were all in the direction suggesting that earlier intervention was associated with higher scores on the PedsQL. As shown in Table 3, earlier intervention was also significantly associated with better pragmatic language and structural language abilities, which in turn were associated with better scores on the PedsQL. 

In a similar vein, higher nonverbal IQ was significantly associated with higher pragmatic language and structural language abilities, and better total score, psychosocial summary score, and school functioning score on the PedsQL. The effect of nonverbal IQ became nonsignificant after allowing for the effects of pragmatic language skills and functional auditory skills on PedsQL. 

### 4.3. Summary and Clinical Implications

Parent-reported functional hearing in everyday situations (PEACH) and pragmatic language skills (GCC) were significant predictors of behavior and emotion (SDQ total and subscale scores) and health-related quality of life (PedsQL total and subscale scores). Psychosocial functioning (encompassing social and school functioning) or the ability to engage socially involves understanding and responding to what’s going on in a social situation, skills which are sampled by the PEACH and the GCC. Our findings support the association of structural language skills with psychosocial health and behavior [2,58], but extend the relationship to functional hearing and pragmatic use of language in everyday settings. We showed that assessments of pragmatic skills, which are founded on structural language skills, have greater relevance than do tests of structural language abilities for identifying DHH children who may be at risk for psychosocial well-being and QOL. 

The present study lends support to the need for early intervention programs to focus on the encouragement of speech and language development [59], and highlights the unmet need for intervention that is targeted on development of functional hearing abilities and pragmatic language skills to support psychosocial health and well-being. While the behavior and emotion outcomes and QOL of this sample of DHH children who received early intervention were rated to be within the normal range on average, the higher-than-normal proportion of those children who were at risk of poor psychosocial well-being and QOL calls for action. The associations reported here suggest that the PEACH scale could be used to effectively identify children who may be at risk at a young age so that they can be supported. An increase in child-directed parent talk at preschool age may facilitate the development of pragmatic skills [60]. Strategies to facilitate the development of pragmatic skills [61] and social skills training [62] can be beneficial for supporting effective functioning in schools. 

Health-care providers can play an important role in the identification of pragmatic and auditory functional performance difficulties and refer those at risk for assessment and intervention services. It is important to establish collaborative relationships with school- and community-based allied health professionals and school counselors to provide adequate referral and support services. Audiological care professionals play a critical role in identifying the diverse listening needs of DHH children in their home and school environments and in providing appropriate levels of technology to meet those needs. In addition, interventions and support that focus on the development of social communication skills and listening abilities are important for children’s psychosocial well-being and for preventing mental health problems.

### 4.4. Strengths and Limitations

This study was the first to examine functional language use (auditory performance, speech intelligibility, and pragmatics) and knowledge of morphosyntactic rules and vocabulary for concurrent associations with psychosocial functioning and QOL in a cohort of DHH children. This study found that functional auditory performance and pragmatic language skills, rather than structural language skills, were significant predictors of psychosocial and QOL outcomes. These results increase understanding about the critical role of functional hearing and use of language for psychosocial well-being, which has direct implications for clinical practice. 

This study was based on parent-reported assessments of psychosocial functioning and QOL. Future work will aim to triangulate evidence from teachers, parents, and the children themselves. Future work will also investigate longitudinal relationships to examine changes over time and to increase understanding about the extent to which early functional hearing abilities may impact later psychosocial well-being and QOL in DHH children using hearing aids or cochlear implants.

We note that there are additional factors (e.g., vestibular disorders, comorbidities, and surgical techniques in cochlear implantation [63]) that might also influence quality of life outcomes that have yet to be investigated.

## 5. Conclusions

This study found that on average, DHH children achieved scores within one SD of population mean for parent-reported behavior and emotion and health-related quality of life at 9 years of age. However, about 7–9% of children demonstrated clinically significant difficulties in peer problems and hyperactivity, and about 6% were found to be at risk of poor psychosocial health; relative to the 2.3% expected in the normal population. Functional hearing ability and pragmatic language skills, rather than structural language skills, were significant predictors of psychosocial and QOL outcomes. These novel findings highlight the importance of targeted interventions for improving functional hearing skills and social communication abilities in DHH children. These findings also emphasize the importance of collaborative approaches among medical, hearing healthcare, audiology, allied health, and educational professionals to identify those at risk so that timely referral and intervention can be implemented for improving psychosocial health and well-being in DHH children.

## Figures and Tables

**Table 1:** Demographic characteristics of participants.

Characteristics		No. of Participants (%)
Sex		
	Female	74 (51.4%)
	Male	70 (48.6%)
Hearing device		
	Hearing aid (HA)	102 (70.8%)
	Cochlear implant (CI)	42 (29.2%)
Degree of hearing loss		
	≤40 dB HL (mild)	33 (22.9%)
	41–60 dB HL (moderate)	50 (34.7%)
	61–80 dB HL (severe)	18 (12.5%)
	≥80 dB HL (profound)	43 (29.9%)
Age at first HA fitting (months)		
	Mean (SD)	11.7 (11.1)
	Median	6.2
	Interquartile range	2.9–20.3
Age at first CI (months)		
	Mean (SD)	26.2 (17.4)
	Median	20.5
	Interquartile range	12.5–38.1
Primary mode of communication		
	Oral only	132 (91.7%)
	Mixed (spoken and sign-supported)	12 (8.3%)
Education setting		
	Mainstream	123 (85.4%)
	Special	5 (3.5%)
	Unknown	16 (11.1%)
Maternal education		
	University or Diploma	62 (43.1%)
	Diploma/certificate	46 (31.9%)
	12 years or less of schooling	36 (25.0%)

**Table 2:** Outcomes of children.

	Measure	Scale	Normative	All Participants	HA (*n* = 102)	CI (*n* = 42)
			Mean (SD)	Mean (SD)	Mean (SD)	Mean (SD)
Nonverbal IQ						
	WNV	Full scale	100 (15)	99.9 (13.5)	99.1 (14.8)	101.6 (9.8)
Language						
	CELF-4	ReLang	100 (15)	84.3 (15.0)	83.9 (15.3)	85.1 (14.5)
		ExLang	100 (15)	84.6 (20.0)	85.0 (19.7)	83.6 (20.8)
Functional auditory performance						
	PEACH	Total score @	87.0 (13.2)	78.4 (13.1)	76.4 (13.6)	83.1 (10.7)
Pragmatic use of language						
	CCC-2	Global composite score #	>45	57.5 (25.0)	56.7 (25.1)	59.6 (25.0)
Speech intelligibility rating						
	SIR	Rating ^$^	1.1 (0.3)	1.6 (0.8)	1.6 (0.9)	1.5 (0.8)
Quality of life						
	PedsQL	Total ^^^	81.3 (15.9)	76.5 (14.4)	75.5 (14.6)	78.8 (13.7)
		Physical ^^^	83.3 (20.0)	88.8 (18.3)	88.3 (17.8)	90.0 (19.6)
		Psychosocial ^^^	80.2 (15.8)	74.8 (15.7)	73.6 (16.3)	77.8 (14.0)
		Emotional ^^^	80.3 (17.0)	72.9 (18.3)	73.0 (18.5)	72.7 (17.9)
		Social ^^^	82.2 (20.1)	78.2 (20.2)	77.2 (21.7)	80.8 (16.1)
		School ^^^	76.9 (20.2)	73.2 (18.7)	70.5 (18.8)	79.8 (16.8)
Behavior and emotion						
	SDQ	Total *		−0.12 (1.0)	−0.20 (1.0)	0.07 (0.9)
		Emotion *		0.25 (0.98)	0.27 (1.0)	0.23 (1.0)
		Conduct *		−0.10 (1.0)	−0.18 (1.1)	0.10 (0.9)
		Hyperactivity *		−0.29 (1.1)	−0.37 (1.1)	−0.09 (1.0)
		Peer Problem *		−0.22 (1.0)	−0.31 (1.0)	0.01 (1.1)
		Prosocial *		0.02 (1.0)	−0.11 (1.0)	0.32 (0.9)

WNV = Wechsler Nonverbal Scale of Ability [29]; CELF-4 = Clinical Evaluation of Language Fundamentals [28]; PEACH = Parents Evaluation of Aural/oral functional performance of Children [21]; CCC-2 = Children’s Communication Checklist [30]; SIR = Speech Intelligibility Index [35,36]; PedsQL = Pediatric Quality of Life Inventory [42]; SDQ = Strengths and Difficulties Questionnaire [38]. @ Normative mean from data of control sample collected as part of the LOCHI study; # cut-off score at the 5th percentile [30]; ^$^ normative mean from data of control sample collected as part of the LOCHI study; ^^^ published normative data [42]; * SDQ scores are shown as z-scores, using age- and gender-appropriate normative data [41].

**Table 3:** Correlations among predictor variables and outcomes measures.

	AgeFit	BE4FA	IQ	PEACH	SIR	GCC	ReLang	ExLang	PHY	EMOF	SOCF	SCHF	PSYSOC	QOLT	EMO	CON	HYP	PEER	PROS	SDQT
**AgeFit**	1.00																			
**BE4FA**	**−0.17**	1.00																		
**IQ**	−0.07	0.08	1.00																	
**PEACH**	−0.10	0.14	0.16	1.00																
**SIR**	0.11	−0.05	−0.28 **	**−0.18**	1.00															
**GCC**	**−0.17**	0.04	0.28 **	0.52 **	−0.37 **	1.00														
**ReLang**	**−0.19**	−0.04	0.54 **	**0.20**	−0.52 **	0.49 **	1.00													
**ExLang**	**−0.20**	−0.06	0.41 **	0.28 *	−0.50 **	0.61 **	0.84 **	1.00												
**PHY**	0.05	0.06	0.16	0.28 *	**−0.18**	0.32 **	**0.18**	**0.21**	1.00											
**EMOF**	0.01	0.06	0.15	0.32 **	−0.04	**0.19**	0.09	0.08	0.34 **	1.00										
**SOCF**	−0.04	0.07	0.15	0.40 **	−0.15	0.39 **	**0.18**	**0.18**	0.56 **	0.51 **	1.00									
**SCHF**	−0.08	0.23 **	0.22 **	0.47 **	**−0.16**	0.49 **	0.29 **	0.31 **	0.52 **	0.44 **	0.60 **	1.00								
**PSYSOC**	−0.04	0.15	0.21	0.48 **	−0.14	0.43 **	0.23 *	**0.23**	0.57 **	0.78 **	0.86 **	0.82 **	1.00							
**QOLT**	−0.01	0.12	**0.21**	0.45 **	**−0.18**	0.44 **	0.24 *	**0.25**	0.83 **	0.65 **	0.84 **	0.80 **	0.93 **	1.00						
**EMO**	−0.02	0.05	0.09	0.33 **	−0.09	0.29 **	0.09	0.10	0.30 **	0.64 **	0.34 **	0.42 **	0.56 **	0.50 **	1.00					
**CON**	−0.03	**0.17**	0.25 **	0.37 **	−0.11	0.29 **	**0.21**	**0.22**	0.25 *	0.36 **	0.35 **	0.41 **	0.45 **	0.41 **	0.28 *	1.00				
**HYP**	−0.01	0.16	0.34 **	0.35 **	−0.15	0.46 **	0.26 *	**0.27**	**0.19**	0.30 **	0.31 **	0.61 **	0.49 **	0.42 **	0.35 **	0.46 **	1.00			
**PEER**	−0.09	0.14	0.15	0.34 **	−0.23 *	0.36 **	0.23 *	**0.25**	0.37 **	0.42 **	0.66 **	0.47 **	0.63 **	0.60 **	0.36 **	0.52 **	0.37 **	1.00		
**PROS**	**−0.20**	0.22 **	**0.19**	0.37 **	**−0.18**	0.34 **	0.12	**0.22**	**0.17**	0.20 *	0.25 *	0.30 **	0.30 **	0.28 *	**0.19**	0.53 **	0.32 **	0.43 **	1.00	
**SDQT**	−0.05	**0.18**	0.29 **	0.47 **	**−0.20**	0.49 **	0.27 *	0.29 **	0.37 **	0.58 **	0.56 **	0.66 **	0.73 **	0.65 **	0.67 **	0.73 **	0.79 **	0.75 **	0.49 **	1.00

**Bold** fonts mark significance at *p* < 0.05. * Depicts significance at *p* < 0.01; ** depicts significance at *p* < 0.001. AgeFit = age at intervention; BE4FA = Better Ear Four frequency average hearing level (average of pure-tone hearing threshold levels at 0.5, 1, 2, and 4 kHz); IQ = nonverbal IQ; PEACH = functional hearing score; SIR = Speech Intelligibility Rating; GCC = General Communication Composite, pragmatic language score; ReLang = receptive language score of CELF-4; ExLang = expressive language score of CELF-4; PHY = physical health score of PedsQL; EMOF = emotional functioning score of PedsQL; SOCF = social functioning score of PedsQL; SCHF = school functioning score of PedsQL; PSYSOC = psychosocial health summary score of PedsQL; QOLT = total score of PedsQL; EMO = emotional difficulties score of Strengths and Difficulties Questionnaire or SDQ; CON = conduct score of SDQ; HYP = hyperactivity score of SDQ; PEER = peer problems score of SDQ; PROS = prosocial behavior score of SDQ; SDQT = total difficulties score of SDQ. Coefficients to the left of the vertical line in the matrix depict intercorrelations of SDQ and PedsQL.

**Table 4:** Multiple regression models using total, emotion, conduct, hyperactivity, peer problems, and prosocial scores of the SDQ as dependent variables.

	SDQT			EMO			CON			HYP			PEER			PROS		
	Est	(95% CI)	*p*-Value	Est	(95% CI)	*p*-Value	Est	(95% CI)	*p*-Value	Est	(95% CI)	*p*-Value	Est	(95% CI)	*p*-Value	Est	(95% CI)	*p*-Value
**AgeFit**	0.05	(−0.09, 0.20)	0.45	0.02	(−0.14, 0.18)	0.84	0.05	(−0.11, 0.21)	0.51	0.08	(−0.07, 0.23)	0.27	0.001	(−0.16, 0.16)	0.99	−0.13	(−0.29, 0.02)	0.09
**BE4FA**	**0.47**	(0.16, 0.78)	**0.003**	**0.45**	(0.10, 0.80)	**0.01**	**0.42**	(0.07, 0.76)	**0.018**	**0.33**	(0.01, 0.66)	**0.04**	0.20	(−0.15, 0.55)	0.26	0.22	(−0.12, 0.56)	0.20
**Device**	**0.40**	(0.08, 0.71)	**0.01**	**0.52**	(0.15, 0.88)	**0.005**	0.33	(−0.13, 0.68)	0.07	0.24	(−0.09, 0.57)	0.16	0.11	(−0.25, 0.47)	0.55	0.09	(−0.26, 0.44)	0.61
**IQ**	0.13	(−0.03, 0.30)	0.11	−0.01	(−0.19, 0.18)	0.95	0.14	(−0.04, 0.32)	0.14	**0.23**	(0.05, 0.40)	**0.01**	0.001	(−0.18, 0.19)	0.99	0.15	(−0.03, 0.33)	0.09
**ReLg**	0.13	(−0.15, 0.42)	0.36	0.17	(−0.16, 0.50)	0.31	0.11	(−0.21, 0.44)	0.48	0.05	(−0.12, 0.22)	0.75	0.07	(−0.24, 0.40)	0.65	−0.32	(−0.63, 0.00)	0.05
**ExpLg**	−0.13	(−0.41, 0.16)	0.38	−0.25	(−0.57, 0.07)	0.13	−0.01	(−0.33, 0.30)	0.94	−0.06	(−0.36, 0.24)	0.68	−0.03	(−0.36, 0.29)	0.84	0.22	(−0.09, 0.53)	0.16
**GCC**	**0.29**	(0.10, 0.49)	**0.004**	0.19	(−0.03, 0.41)	0.09	0.06	(−0.16, 0.27)	0.6	**0.35**	(0.15, 0.55)	**<0.001**	0.20	(−0.03, 0.42)	0.08	0.14	(−0.07, 0.36)	0.19
**PEACH**	**0.33**	(0.17, 0.50)	**<0.001**	**0.31**	(0.12, 0.50)	**0.002**	**0.32**	(0.14, 0.51)	**<0.001**	0.17	(−0.01, 0.34)	0.06	**0.21**	(0.02, 0.40)	**0.03**	**0.23**	(0.05, 0.41)	**0.01**
**SIR**	−0.01	(−0.18, 0.15)	0.87	−0.04	(−0.22, 0.15)	0.69	0.04	(−0.14, 0.22)	0.65	0.05	(−0.12, 0.22)	0.55	0.11	(−0.25, 0.47)	0.55	−0.08	(−0.26, 0.09)	0.36
**Adj *R*^2^**	**0.332**			**0.129**			**0.166**			**0.262**			**0.135**			**0.194**		

**Bold** values depict significance at *p* < 0.05. Est = estimate (beta); 95% CI = 95% confidence interval of parameter estimate; SDQT = Total Difficulties score of Strengths and Difficulties Questionnaire or SDQ; EMO = emotional difficulties score of SDQ; CON = conduct score of SDQ; HYP = hyperactivity score of SDQ; PEER = peer problems score of SDQ; PROS = prosocial behavior score of SDQ; AgeFit = age at intervention; BE4FA = Better Ear Four frequency average hearing level (average of hearing threshold level at 0.5, 1, 2, and 4 kHz); Device = hearing aids or cochlear implants; IQ = nonverbal IQ; ReLang = receptive language score of CELF-4; ExLang = expressive language score of CELF-4; GCC = General Communication Composite, pragmatic language score; PEACH = functional hearing score; SIR = Speech Intelligibility Rating; Adj *R*^2^ = adjusted *R*^2^.

**Table 5:** Multiple regression models using total, psychosocial health, school functioning, social functioning, emotional functioning and physical health scores of the Pediatric Quality of Life Inventory (PedsQL) as dependent variables.

	QOLT			PSYSOC			SCHF			SOCF			EMOF			PHY		
	Est	(95% CI)	*p*-Value	Est	(95% CI)	*p*-Value	Est	(95% CI)	*p*-Value	Est	(95% CI)	*p*-Value	Est	(95% CI)	*p*-Value	Est	(95% CI)	*p*-Value
**AgeFit**	0.08	(−0.06, 0.23)	0.26	0.05	(−0.10, 0.20)	0.52	0.05	(−0.10, 0.19)	0.51	0.03	(−0.13, 0.19)	0.7	0.04	(−0.13, 0.20)	0.67	0.12	(−0.05, 0.28)	0.16
**BE4FA**	0.29	(−0.03, 0.62)	0.08	**0.34**	(0.02, 0.66)	**0.04**	0.28	(−0.03, 0.60)	0.08	0.11	(−0.23, 0.46)	0.52	**0.46**	(0.10, 0.82)	**0.01**	0.22	(−0.14, 0.58)	0.22
**Device**	0.25	(−0.08, 0.59)	0.14	0.29	(−0.04, 0.62)	0.09	0.1	(−0.22, 0.42)	0.53	0.11	(−0.25, 0.46)	0.55	**0.52**	(0.15, 0.88)	**0.006**	0.2	(−0.17, 0.57)	0.28
**IQ**	0.06	(−0.12, 0.23)	0.53	0.05	(−0.12, 0.22)	0.56	0.02	(−0.15, 0.19)	0.82	0.03	(−0.15, 0.21)	0.74	0.08	(−0.11, 0.27)	0.42	0.05	(−0.14, 0.24)	0.63
**ReLg**	0.16	(−0.15, 0.46)	0.31	0.21	(−0.10, 0.51)	0.18	0.2	(−0.10, 0.49)	0.18	0.12	(−0.21, 0.44)	0.47	0.20	(−0.13, 0.53)	0.23	0.03	(−0.30, 0.37)	0.84
**ExpLg**	−0.14	(−0.44, 0.16)	0.36	−0.19	(−0.48, 0.11)	0.21	−0.07	(−0.36, 0.22)	0.63	−0.17	(−0.49, 0.14)	0.28	−0.22	(−0.55, 0.10)	0.18	−0.01	(−0.34, 0.32)	0.93
**GCC**	**0.27**	(0.06, 0.48)	**0.01**	**0.25**	(0.05, 0.46)	**0.02**	**0.30**	(0.10, 0.50)	**0.003**	**0.28**	(0.06, 0.50)	**0.01**	0.03	(−0.19, 0.26)	0.78	0.21	(−0.02, 0.43)	0.07
**PEACH**	**0.33**	(0.16, 0.51)	**<0.001**	**0.38**	(0.20, 0.55)	**<0.001**	**0.29**	(0.12, 0.46)	**<0.001**	**0.28**	(0.09, 0.46)	**0.004**	**0.370**	(0.18, 0.57)	**0.004**	0.180	(−0.02, 0.37)	0.080
**SIR**	−0.01	(−0.19, 0.16)	0.87	0.02	(−0.14, 0.19)	0.78	0.07	(−0.10, 0.23)	0.42	−0.02	(−0.20, 0.16)	0.82	0.01	(−0.17, 0.20)	0.88	−0.07	(−0.26, 0.12)	0.45
**Adj *R*^2^**	**0.249**			**0.268**			**0.308**			**0.160**			**0.114**			**0.094**		

**Bold** values depict significance at *p* < 0.05. Est = estimate (beta); 95% CI = 95% confidence interval of parameter estimate; QOLT = total score of Pediatric Quality of Life Inventory or PedsQL; PSYSOC = psychosocial health score of PedsQL; SCHF = school functioning score of PedsQL; SOCF = social functioning score of PedsQL; EMOF = emotional functioning score of PedsQL; PHY = physical health score of PedsQL; AgeFit = age at intervention; BE4FA = Better Ear Four frequency average hearing level (average of hearing threshold level at 0.5, 1, 2, and 4 kHz); Device = hearing aids or cochlear implants; IQ = nonverbal IQ; ReLang = receptive language score of CELF-4; ExLang = expressive language score of CELF-4; GCC = General Communication Composite, pragmatic language score; PEACH = functional hearing score; SIR = Speech Intelligibility Rating. Adj *R*^2^ = adjusted *R*^2^.

## Data Availability

The authors are willing to share unidentified data from the study with bona fide researchers who provide a methodologically sound proposal for use in achieving the goals of the approved proposal. The de-identified data can be made available through discussion with the corresponding author and chief investigator (T.Y.C.C.), with permission of the study chief investigator team, and approval of the Institutional Review Board. As a longitudinal study that is currently in progress, participants have not given permission for data to be available to people not directly involved in this research.

## References

[1] Castellanos I., Kronenberger W.G., Pisoni D.B. (2018). Psychosocial Outcomes in Long-Term Cochlear Implant Users. Ear Hear..

[2] Dammeyer J. (2010). Psychosocial development in a Danish population of children with cochlear implants and deaf and hard-of-hearing children. J. Deaf. Stud. Deaf. Educ..

[3] Fellinger J., Holzinger D., Beitel C., Laucht M., Goldberg D.P. (2009). The impact of language skills on mental health in teenagers with hearing impairments. Acta Psychiatr. Scand..

[4] Overgaard K.R., Oerbeck B., Wagner K., Friis S., Ohre B., Zeiner P. (2021). Youth with hearing loss: Emotional and behavioral problems and quality of life. Int. J. Pediatr. Otorhinolaryngol..

[5] Stika C.J., Eisenberg L.S., Carter A.S., Johnson K.C., Hammes Ganguly D.M., Henning S.C., DesJardin J.L. (2021). Developmental Outcomes in Early-Identified Children Who Are Hard of Hearing at 2 to 3 Years of Age. Ear Hear..

[6] Zaidman-Zait A., Most T. (2020). Pragmatics and Peer Relationships Among Deaf, Hard of Hearing, and Hearing Adolescents. Pediatrics.

[7] Haukedal C.L., von Koss Torkildsen J., Lyxell B., Wie O.B. (2018). Parents’ Perception of Health-Related Quality of Life in Children With Cochlear Implants: The Impact of Language Skills and Hearing. J. Speech Lang. Hear. Res..

[8] Haukedal C.L., Lyxell B., Wie O.B. (2020). Health-Related Quality of Life With Cochlear Implants: The Children’s Perspective. Ear Hear..

[9] Roland L., Fischer C., Tran K., Rachakonda T., Kallogjeri D., Lieu J.E. (2016). Quality of Life in Children with Hearing Impairment: Systematic Review and Meta-analysis. Otolaryngol. Head Neck Surg..

[10] Hofmann M., Meloche M., Zwolan T.A. (2020). Health related quality of life in adolescent cochlear implant users. Cochlear Implants Int..

[11] Martin D., Bat-Chava Y., Lalwani A., Waltzman S.B. (2011). Peer relationships of deaf children with cochlear implants: Predictors of peer entry and peer interaction success. J. Deaf. Stud. Deaf. Educ..

[12] Sarant J.Z., Harris D.C., Galvin K.L., Bennet L.A., Canagasabey M., Busby P.A. (2018). Social Development in Children With Early Cochlear Implants: Normative Comparisons and Predictive Factors, Including Bilateral Implantation. Ear Hear..

[13] Leigh G., Ching T.Y., Crowe K., Cupples L., Marnane V., Seeto M. (2015). Factors Affecting Psychosocial and Motor Development in 3-Year-Old Children Who Are Deaf or Hard of Hearing. J. Deaf. Stud. Deaf. Educ..

[14] World Health Organization (1998). Programme on Mental Health: WHOQOL User Manual.

[15] Wong C.L., Ching T.Y., Leigh G., Cupples L., Button L., Marnane V., Whitfield J., Gunnourie M., Martin L. (2018). Psychosocial development of 5-year-old children with hearing loss: Risks and protective factors. Int. J. Audiol..

[16] Wong C.L., Ching T.Y.C., Cupples L., Button L., Leigh G., Marnane V., Whitfield J., Gunnourie M., Martin L. (2017). Psychosocial Development in 5-Year-Old Children With Hearing Loss Using Hearing Aids or Cochlear Implants. Trends Hear..

[17] Szarkowski A., Toe D. (2020). Pragmatics in Deaf and Hard of Hearing Children: An Introduction. Pediatrics.

[18] American Psychological Association APA Dictionary of Psychology. https://dictionary.apa.org/.

[19] Ireton H. (2005). Child Development Inventory.

[20] Zimmerman I.L., Steiner V.G., Pond R.E. (2002). Preschool Language Scale.

[21] Ching T.Y., Hill M. (2007). The Parents’ Evaluation of Aural/Oral Performance of Children (PEACH) scale: Normative data. J. Am. Acad. Audiol..

[22] Freeman V., Pisoni D.B., Kronenberger W.G., Castellanos I. (2017). Speech Intelligibility and Psychosocial Functioning in Deaf Children and Teens with Cochlear Implants. J. Deaf. Stud. Deaf. Educ..

[23] Reynolds C.R., Kamphaus R.W. (2004). Behavior Assessment System for Children Manual.

[24] Kushalnagar P., Topolski T.D., Schick B., Edwards T.C., Skalicky A.M., Patrick D.L. (2011). Mode of communication, perceived level of understanding, and perceived quality of life in youth who are deaf or hard of hearing. J. Deaf. Stud. Deaf. Educ..

[25] Most T. (2007). Speech intelligibility, loneliness, and sense of coherence among deaf and hard-of-hearing children in individual inclusion and group inclusion. J. Deaf. Stud. Deaf. Educ..

[26] Zaidman-Zait A., Dotan A. (2017). Everyday Stressors in Deaf and Hard of Hearing Adolescents: The Role of Coping and Pragmatics. J. Deaf. Stud. Deaf. Educ..

[27] Ching T.Y., Leigh G., Dillon H. (2013). Introduction to the longitudinal outcomes of children with hearing impairment (LOCHI) study: Background, design, sample characteristics. Int. J. Audiol..

[28] Semel E., Wiig E.H., Secord W. (2003). Clinical Evaluation of Language Fundamentals.

[29] Wechsler D., Naglieri J. (2006). Wechsler Nonverbal Scale of Ability: WNV.

[30] Bishop D.V. (2003). The Children’s Communication Checklist.

[31] Norbury C.F., Nash M., Baird G., Bishop D. (2004). Using a parental checklist to identify diagnostic groups in children with communication impairment: A validation of the Children’s Communication Checklist–2. Int. J. Lang. Commun. Disord..

[32] Ching T.Y., Hill M., Dillon H. (2008). Effect of variations in hearing-aid frequency response on real-life functional performance of children with severe or profound hearing loss. Int. J. Audiol..

[33] Bagatto M.P., Scollie S.D. (2013). Validation of the Parents’ Evaluation of Aural/Oral Performance of Children (PEACH) Rating Scale. J. Am. Acad. Audiol..

[34] Golding M., Pearce W., Seymour J., Cooper A., Ching T., Dillon H. (2007). The relationship between obligatory cortical auditory evoked potentials (CAEPs) and functional measures in young infants. J. Am. Acad. Audiol..

[35] Nikolopoulos T.P., Archbold S.M., Gregory S. (2005). Young deaf children with hearing aids or cochlear implants: Early assessment package for monitoring progress. Int. J. Pediatr. Otorhinolaryngol..

[36] Allen C., Nikolopoulos T.P., Dyar D., O’Donoghue G.M. (2001). Reliability of a rating scale for measuring speech intelligibility after pediatric cochlear implantation. Otol. Neurotol..

[37] Yoshinaga-Itano C. (2000). Successful outcomes for deaf and hard-of-hearing children. Semin. Hear..

[38] Goodman R. (1997). The Strengths and Difficulties Questionnaire: A research note. J. Child Psychol. Psychiatry.

[39] Niclasen J., Dammeyer J. (2016). Psychometric Properties of the Strengths and Difficulties Questionnaire and Mental Health Problems Among Children With Hearing Loss. J. Deaf. Stud. Deaf. Educ..

[40] Hintermair M. (2007). Prevalence of socioemotional problems in deaf and hard of hearing children in Germany. Am. Ann. Deaf..

[41] Mellor D. (2005). Normative data for the Strengths and Difficulties Questionnaire in Australia. Aust. Psychol..

[42] Varni J.W., Seid M., Kurtin P.S. (2001). PedsQL 4.0: Reliability and validity of the Pediatric Quality of Life Inventory version 4.0 generic core scales in healthy and patient populations. Med. Care.

[43] Varni J.W., Burwinkle T.M., Seid M., Skarr D. (2003). The PedsQL 4.0 as a pediatric population health measure: Feasibility, reliability, and validity. Ambul. Pediatr..

[44] Wang J., Quach J., Sung V., Carew P., Edwards B., Grobler A., Gold L., Wake M. (2019). Academic, behavioural and quality of life outcomes of slight to mild hearing loss in late childhood: A population-based study. Arch. Dis. Child.

[45] Wake M., Tobin S., Cone-Wesson B., Dahl H.H., Gillam L., McCormick L., Poulakis Z., Rickards F.W., Saunders K., Ukoumunne O.C. (2006). Slight/mild sensorineural hearing loss in children. Pediatrics.

[46] StatSoft Inc. (2011). Statistica Software.

[47] Ching T.Y.C., Dillon H., Button L., Seeto M., Van Buynder P., Marnane V., Cupples L., Leigh G. (2017). Age at Intervention for Permanent Hearing Loss and 5-Year Language Outcomes. Pediatrics.

[48] Stevenson J., McCann D., Watkin P., Worsfold S., Kennedy C., Team H.O.S. (2010). The relationship between language development and behaviour problems in children with hearing loss. J. Child Psychol. Psychiatry.

[49] Theunissen S.C., Rieffe C., Netten A.P., Briaire J.J., Soede W., Schoones J.W., Frijns J.H. (2014). Psychopathology and its risk and protective factors in hearing-impaired children and adolescents: A systematic review. JAMA Pediatr..

[50] Stevenson J., Kreppner J., Pimperton H., Worsfold S., Kennedy C. (2015). Emotional and behavioural difficulties in children and adolescents with hearing impairment: A systematic review and meta-analysis. Eur. Child Adolesc. Psychiatry.

[51] Dye M.W., Bavelier D. (2010). Attentional enhancements and deficits in deaf populations: An integrative review. Restor. Neurol. Neurosci..

[52] Corina D., Singleton J. (2009). Developmental social cognitive neuroscience: Insights from deafness. Child Dev..

[53] Cummings L. (2014). Pragmatic Disorders.

[54] Borton S.A., Mauze E., Lieu J.E. (2010). Quality of life in children with unilateral hearing loss: A pilot study. Am. J. Audiol..

[55] Lovett R.E., Kitterick P.T., Hewitt C.E., Summerfield A.Q. (2010). Bilateral or unilateral cochlear implantation for deaf children: An observational study. Arch. Dis. Child.

[56] Umansky A.M., Jeffe D.B., Lieu J.E. (2011). The HEAR-QL: Quality of life questionnaire for children with hearing loss. J. Am. Acad. Audiol..

[57] Yu C.Y., Jeffe D.B., Kenna M.A., Germiller J.A., Lieu J.E.C. (2021). Validation of a Parent Proxy Quality-of-Life Measure for Young Children With Hearing Loss. Laryngoscope.

[58] Netten A.P., Rieffe C., Theunissen S.C., Soede W., Dirks E., Korver A.M., Konings S., Oudesluys-Murphy A.M., Dekker F.W., Frijns J.H. (2015). Early identification: Language skills and social functioning in deaf and hard of hearing preschool children. Int. J. Pediatr. Otorhinolaryngol..

[59] Joint Committee on Infant Hearing (2019). Year 2019 Position Statement: Principles and Guidelines for Early Hearing Detection and Intervention Programs. J. Early Hear. Detect. Interv..

[60] Yoshinaga-Itano C., Sedey A.L., Mason C.A., Wiggin M., Chung W. (2020). Early Intervention, Parent Talk, and Pragmatic Language in Children With Hearing Loss. Pediatrics.

[61] Goberis D., Beams D., Dalpes M., Abrisch A., Baca R., Yoshinaga-Itano C. (2012). The missing link in language development of deaf and hard of hearing children: Pragmatic language development. Semin. Speech Lang..

[62] Antia S.D., Kreimeyer K.H. (1997). The generalization and maintenance of the peer social behaviors of young children who are deaf or hard of hearing. Lang. Speech Hear. Serv. Sch..

[63] Freni F., Gazia F., Slavutsky V., Scherdel E.P., Nicenboim L., Posada R., Portelli D., Galletti B., Galletti F. (2020). Cochlear Implant Surgery: Endomeatal Approach versus Posterior Tympanotomy. Int. J. Environ. Res. Public Health.

